# AI-driven analysis by identifying risk factors of VL relapse in HIV co-infected patients

**DOI:** 10.1038/s41598-025-07406-7

**Published:** 2025-07-01

**Authors:** Abhishek Kumar, Sanchita Mondal, Debnarayan Khatua, Debashree Guha, Budhaditya Mukherjee, Arista Lahiri, Dilip K. Prasad, Arif Ahmed Sekh

**Affiliations:** 1 School of Medical Science and Technology, IIT Kharagpur, Kharagpur, West Bengal 721302 India; 2 Department of Mathematics and Statistics, Vignan’s Foundation for Science, Technology and Research, Andhra Pradesh, 522213 India; 3 Dr B C Roy Multi Speciality Medical Research Centre, IIT Kharagpur, Kharagpur, West Bengal 721302 India; 4 Department of Computer Science, UiT The Arctic University of Norway, Tromsø, 9017 Norway

**Keywords:** VL relapse, Relapse prediction, Risk factors for VL relapse, Cox regression, Explainable machine learning, Immunological disorders, Infectious diseases

## Abstract

Visceral Leishmaniasis (VL), also known as Kala-Azar, poses a significant global public health challenge and is a neglected disease, with relapses and treatment failures leading to increased morbidity and mortality. This study introduces an explainable machine learning approach to predict VL relapse and identify critical risk factors, thereby aiding patient monitoring and treatment strategies. Leveraging data from a follow-up study of 571 patients, the survival machine learning models are applied, including Random Survival Forest (RSF), Survival Support Vector Machine (SSVM), and eXtreme Gradient Boosting (XGBoost), for relapse prediction. The results demonstrated that RSF, with a C-index of 0.85, outperformed the conventional Cox Proportional Hazard (CPH) model (C-index 0.8), offering improved prediction capabilities by capturing non-linear relationships and variable interactions. To address the lack of transparency (in terms of feature importance) in Machine Learning (ML) models, the SHapley Additive exPlanation (SHAP) method is employed, which enhances model interpretability (feature importance) through visual insights. SHAP dependence plots allowed the healthcare professionals to evaluate which factors encourage the occurrence of the relapse. A statistically significant relationship between HIV co-infection (HR=3.92, 95% CI=2.03–7.58) and VL relapse was identified through -2 log-likelihood ratio and chi-square tests. These results indicate the promise of explainable artificial intelligence (XAI) for making clinical decisions and remedying recurrences in VL.

## Introduction

VL, a parasitic disease and a neglected disease^1^, has become a serious public health concern in tropical and subtropical regions of developing countries with high population densities, such as India^2^, Brazil^3^, and others^4^. In the Indian subcontinent (ISC), the protozoan parasite *L. donovani* and/or *L. infantum* causes the disease of VL, also known as Post-Kala-Azar-Dermal-Leishmaniasis (PKDL), which is contracted by biting an infected sand fly^5^, *Phlebotomus argentipes*^6^. More precisely, more than 10K cases are reported annually (in India, globally an estimated 500,000 new cases of VL and more than 50,000 deaths from the disease each year^7^), with the majority of these cases occurring in the state of Bihar in India^8^. As VL has a concentrated distribution that is catastrophic for affected populations, these numbers may not accurately reflect the full societal effect of this disease. However, the disease is highly neglected because it is characterized by the formation of painless skin lesions, which many people do not report. Another significant complication associated with this disease is the presence of HIV infection along with VL or PKDL infections^9^, as co-infections increase the rate of transmission of the parasite and mortality among patients^10^. Therefore, it is essential to account for the factors that influence the mortality rate in patients with VL infection^11^.

Statistical analysis has been performed to quantify relapse occurrences and identify clinical and laboratory indicators relevant to individuals co-infected with Leishmania and HIV and other diseases^12^. The conventional Cox regression model was used to discern potential associations and prognostic factors in the context of Leishmania-HIV co-infection. The scientific community has extensively embraced the traditional CPH model due to its quick computing time, intuitive interface, and, most importantly, its accurate results^13,14^. However, the model has several inherent drawbacks. This model needs to be revised in high-dimensional contexts, for example, where the number of features exceeds the number of data instances. In addition, the proportionality of the hazard functions for any two patients, i.e., the ratio that remains constant throughout time, and the uncorrelated characteristics are two more restricted assumptions that CPH regression predicates. Above all, though, it can not accurately predict non-linearity and interaction effects, which are frequently found in censored data.

In recent years, ML has been shown to be a valuable adjunct to conventional statistical methods in terms of improving diagnosis, prognosis, and prediction in the clinical domain^15–17^. As ML methods are more flexible and can capture non-linearity and variable interactions, they have been effectively adapted to handle medical datasets. This has expanded its utilization for the analysis of the patient’s outcome. To address the aforementioned limitation in CPH analysis, ML techniques have been utilized in the study. In this context, several studies have repeatedly shown that ML methods can predict patient outcomes at least as well as conventional CPH analysis^18,19^. However, ML models are considered black boxes in this research because it makes it difficult to understand how a model came to its predictions. More significantly, this reduces the trustworthiness of physicians and patients in the prediction of ML models. To overcome the facts above, explainable machine learning approaches seek to offer an understandable explanation of how an ML model achieved its results^20^. Furthermore, it can help confirm certain qualities that are crucial for ML models, such as robustness and fairness^21^. An explainable ML model is particularly critical in the clinical domain; the probable reason behind this is to make decisions based on model predictions. Users must be able to comprehend how the model arrived at that decision to trust and, more crucially, accept the prediction model.

Although XAI is used in various clinical applications, such as heart failure survival^22^, Alzheimer’s disease diagnosis^23^, lung cancer detection^24^, etc. However, researchers also argue about the usability and trust of state-of-the-art XAI methods applicable to healthcare. Ghassemi et al.^25^ explain the impact of model and data complexity. For simple models, explanations can still be confusing due to hidden factors and too much information. For complex models, such as those that analyze images or text, explanations often do not clearly show what influenced the decision, leading to misunderstandings. Additionally, people might wrongly assume that the model’s reasoning matches their own, which can be misleading. AI will significantly impact medicine, but explainability methods currently do not provide reassurance for individual decisions or increase trust in clinical practice. These methods are valuable for model troubleshooting and system audits, helping to improve performance and identify biases. Despite these limitations, AI can revolutionize healthcare with accurate diagnostics and personalized treatments. Healthcare workers should use AI explanations with caution, and regulators should carefully consider the requirements for clinical deployment. Brankovic et al.^26^ suggest that trustworthy explanations can be achieved if: 1) diverse ML methods consistently identify influential relationships, 2) observable input factors can be modified to alter the model output, and 3) the adjusted model outcomes align with real-world results. Without meeting these conditions, no explanation method should be considered reliable or used to guide clinical interventions. However, XAI methods can still be beneficial for clinicians when they have limited information about a patient or need to identify “at-risk” patients who appear stable. In such scenarios, there is a risk of overrelying on artificial intelligence (AI), which could result in incorrect clinical decisions. Cabitza et al.^27^ reveal the ‘XAI halo effect,’ where misleading explanations caused participants to overlook verifying the advice’s accuracy. This highlights a challenge in XAI: even correct advice can lead to misjudgment if explanations are not coherent and contextually relevant. The study highlights the need for explanations to be accurate and relevant, especially when decision accuracy is crucial, questioning the use of explanations if their potential to mislead outweighs their benefits. However, researchers agree that the appropriate use of XAI can lead to the development of a better healthcare system. XAI can be beneficial during model development and can be used as a tool for better understanding AI models.

In this study, XAI is used as a tool to optimize the development of models. Using data from the follow-up study conducted in So Paulo, Brazil^10^, survival ML models, that is, RSF, SSVM, and XGBoost models, are used along with conventional CPH models. Furthermore, the SHAP method is used to interpret the significant relationship between a feature and the relapse outcome provided by the ML models, which enhances the explainability and transparency (feature importance and relation) of the model’s predictions. By computing Shapley values and constructing summary and dependence plots, the aim is to provide insight into how the features participate in decision-making. This transparency and analysis are crucial to fostering trust in the predictive model within clinical and research settings, ultimately helping to develop more effective interventions and pipelines to combat VL infections and their associated complications. In relapse analysis, the calculation of hazard ratios, confidence intervals, and p-values is typically achieved using statistical methods such as CPH analysis. Some studies showed that survival ML models can outperform conventional methods such as Cox regression^28^. However, the study combines ML models and the XAI tool to identify variables that optimize a CPH model by providing robust statistical measures to understand the relationship between risk factors and outcomes.

### Problem statement

Some public health issues can be complicated to tackle, such as Kala-azar, especially in HIV patients, imposing a challenge to traditional health interventions due to its high risk of recurrence and death. The use of traditional statistics, mainly the CPH model in survival analysis, has been prevalent, but they have many inherent weaknesses. These models presuppose that there are proportional hazards, that the effects of variables are linear, and that large-scale data that include complex, poorly controlled interactions are highly problematic. These limitations impede accurate prediction of disease-free survival, especially when dealing with multiple clinical and laboratory risk factors. Because VL progress is time-dependent in persons co-infected with HIV, these people require continued administration of antiretroviral therapy (ART), and better models are needed to cater to the management needs. Recent progress in AI and explainable risk factor identification in health monitoring opened up new possibilities for analysis and predictions. A similar mechanism is not explored in VL, specifically in Leishmania-HIV co-infected Patients.

### Motivation

The primary aim of this study is to address the urgent need for improved secondary prevention and stratification of risk factors for VL relapse in the context of HIV infection. Accurate diagnostic models and risk classification schemes are essential for both scientific understanding and effective patient management to reduce mortality. ML algorithms, including survival models such as RSF, SSVM, and XGBoost, offer significant potential as prognostic tools. Despite their proven benefits in improving patient outcomes, the practical application of ML models remains challenging. This study tackles this issue by employing EXAI techniques, specifically the SHAP method, to interpret ML models. The use of SHAP improves the predictive power of risk models and builds trust among healthcare professionals by providing a balanced approach to secondary VL relapse prevention by identifying important characteristics. Although SHAP has been widely used for the selection of explainable characteristics in various diseases such as Parkinson’s disease^29^, heart disease^30^, and breast cancer^31^, its application in the prediction of relapses of VL has not been explored until now. This study aims to fill that gap, thereby improving prediction accuracy and fostering cautious optimism among healthcare providers.

### Objectives

The objectives of the work are the following.

- To evaluate the performance of survival machine learning models (RSF, SSVM, XGBoost) compared to the traditional Cox Proportional Hazards (CPH) model for predicting VL relapse.
- Identify and rank key clinical and laboratory predictors of relapsed VL (feature selection) using explainable machine learning techniques, particularly SHAP (SHapley additive explanations).
- Optimize the conventional CPH model by integrating critical predictors (features) identified by machine learning to improve the estimation of the hazard ratio and predictive accuracy.

### Background

The intricacies of the dynamics of co-infection, particularly involving HIV-1 and parasites such as Leishmania, have been extensively explored through mathematical modeling and simulation frameworks. Lloyd-Smith et al.^32^ provided a comprehensive foundation for understanding co-infections, including visceral leishmaniasis, within diverse populations. Building on this, I. M. Elmojtaba’s mathematical models underscored the pivotal role of reducing the reproduction number for effective control^32–34^. Transitioning to Ethiopia, Hussaini et al.^35^ delved into the transmission dynamics of VL and HIV and identified key parameters by influencing the comprehensive VL-HIV model. Further insights emerged from studies in Bihar, India, where Cloots et al.^36^ demonstrated the significant impact of VL-HIV+ and PKDL on the incidence of VL at the village level. The mathematical model of Melese and Alemneh^37^ emphasized the necessity of targeted interventions to control co-infection, focusing on enhancing recovery rates and reducing transmission. The genetic diversity of L. donovani in northern Ethiopia, explored by Franssen et al.^38^, shed light on the occurrence of visceral leishmaniasis, and revealed intriguing patterns in parasite relapses and genetic variations within host patients^36–38^. The exists different machine learning algorithms such as Logistic Regression (LR) and XGBoost, applied in other diseases such as heart disease^39,40^ and also Random Forest^41^ and SVM^42^.

Furthermore, Das et al.^43^ conducted a longitudinal study in India, spanning 24 months in the Saran and Muzaffarpur districts of Bihar, to understand the transmission dynamics of VL within households affected by various leishmanial manifestations. The study indicated that households close to the occurrence of VL within a family member exhibited the highest rates of seroconversion. However, contrary to expectations, there was no empirical support for a higher transmission rate in households with PKDL or individuals who tested positive for the rK39 antigen, indicating an active VL. Mathur et al.^44^ extended these insights through a two-year study in northern India, identifying 104 cases of VL or post-kala-azar cutaneous leishmaniasis, with 5.7% coinfection with HIV. In particular, antileishmanial therapy showed limited efficacy in cases of coinfection, revealing a chronic and relapsing course. However, Sinha et al.^45^ explored the outcomes of liposomal amphotericin B treatment followed by combined antiretroviral treatment (cART) for VL-HIV co-infection in Bihar, India; of the 55 included patients, 27 experienced VL relapse during the two-year treatment period. The study highlighted a correlation between CD4 counts and subsequent relapses, with CD4 counts $\le$ 200 cells / L six months after the initiation of cART predictive of relapse. Although amphotericin B intolerance did not interrupt treatment, the study emphasized the frequent occurrence of VL relapse during treatment. These longitudinal studies in Bihar provide crucial information on the dynamics of VL transmission within households, the challenges of co-infection with HIV, and the complexities associated with treatment outcomes, particularly the risk of relapse during cART^43–45^.

XAI is a growing field of research that enables different explanations of decision-making in an AI system. It is widely used in different fields, including healthcare^46^. One such method involved identifying Feature Importance^47^. XAI methods, such as SHAP, leverage Shapley values from cooperative game theory to fairly determine the contribution of each feature (such as biomarkers or clinical attributes) to a healthcare outcome. This approach ensures a transparent and fair analysis of how each feature influences the outcome of the disease, making it a valuable tool in medical research and diagnostics^48^. Gradient-weighted Class Activation Mapping (GradCAM) is a powerful tool in healthcare to interpret deep neural network (CNN) models. Using gradient information from the final convolutional layer, GradCAM generates heat maps that highlight the regions of medical images (such as magnetic resonance or CT scans) that the model considers important for its predictions. This helps clinicians understand and trust model decisions, improves diagnostic accuracy, and facilitates better patient outcomes^49^. Local Interpretable Model-agnostic Explanations (LIME) is used in healthcare to provide clear, instance-level explanations of machine learning model predictions, helping clinicians understand the factors influencing individual patient outcomes. This approach improves transparency and trust in AI-driven decisions, ultimately supporting better patient care and personalized treatment plans^50^. Other XAI methods, such as Explainable Boost Machine (EBM)^51^ and Case-Based Reasoning (CBR) are also applied in various interpretations in healthcare decision-making.

Moreover, the aforementioned literature utilized conventional statistical analysis to understand the complex dynamics of VL transmission, co-infection with HIV, and its treatment outcomes over several longitudinal studies. Simão et al.^10^ also conducted a study in Brazil and used the conventional Cox regression model to identify predictors of relapse in patients with visceral leishmaniasis, with the aim of improving therapeutic strategies and follow-up of patients despite its public health challenges. Using recent advances in artificial intelligence in the proposed approach, explainable machine learning is used to highlight the key predictors of relapse prediction of VL, which improves predictive precision and can help healthcare professionals in this disease prognosis for individual patients in terms of risk prediction.

## Methods

Based on the findings from the previous study^10^, three distinct multivariate models were constructed, each incorporating key predictors identified by the p-value of their hazard ratios. Model 1 integrates all essential clinical parameters, while Model 2 includes all relevant laboratory parameters. Model 3 is a comprehensive model that combines both clinical and laboratory parameters. In particular, the age group and the treatment with liposomal amphotericin were included in all multivariate models, regardless of their significance in the univariate analysis. This inclusion ensures that these variables are taken into account due to their potential clinical importance. Table 1 provides detailed information on the features used in each multivariate model. In this study, three survival ML models, namely RSF, SSVM, and XGBoost, were applied to the aforementioned multivariate models to predict VL relapse. The performance of these ML models was compared with conventional CPH models to identify the best relapse prediction model based on the concordance index (c-index).

**Fig. 1:**
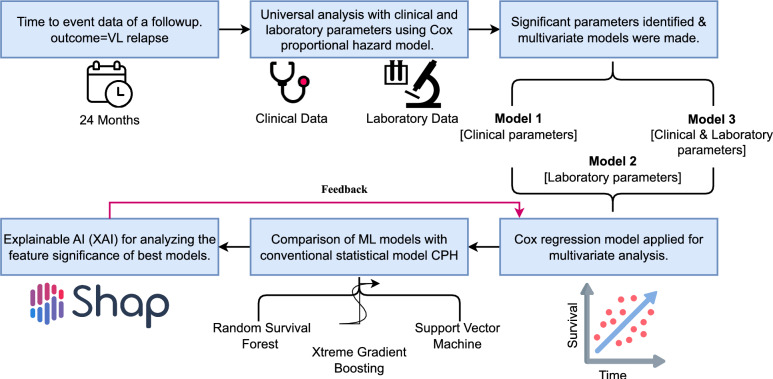
Overview of how the explainable AI method SHAP is utilized to identify risk predictors in the proposed work. First, 24 months of data containing clinical and laboratory data are considered for the analysis. Next, three different models containing clinical only, laboratory, and combined data are utilized for analysis. Next, three different ML models, RSF, XGBoost, and SVM, were applied along with the state-of-the-art CPH model. The ML models, along with the SHAP explanation, are used to optimize the CPH model. Finally, the optimized model is used as the proposed model.

The explainable method SHAP was employed in the best-performing relapse prediction model to identify risk predictors for VL relapse. Shapley values were computed to quantify the impact of individual features on model predictions. These SHAP values were then used to create summary and dependence plots, which visually represent the relationships between features and predicted relapses. The role of explainability is crucial in this context, as it facilitates the interpretation and understanding of the complex decision-making processes inherent in survival ML models by demonstrating the role of each feature. By using the XAI method, SHAP values corresponding to the features and associated visualizations were obtained, providing insights into the underlying mechanisms and the role of each feature driving model predictions. This enhanced transparency and interpretability are essential for ensuring the trustworthiness and usability of predictive models in clinical and research settings. An overview of the relapse prediction process in this study is depicted in Fig.1.

**Table 1:** Parameters in Multivariate Model 1, Model 2, and Model 3.

	Model 1	Model 2	Model 3
Clinical Signs and Symptoms			
HIV Coinfection	$\checkmark$	$\checkmark$	$\checkmark$
Edema of Lower Limbs	$\checkmark$		$\checkmark$
Secondary Pneumonia	$\checkmark$		$\checkmark$
Laboratory Data			
Platelet Count upon Admission		$\checkmark$	$\checkmark$
Hematocrit Change (%)		$\checkmark$	$\checkmark$
White Cell Count Change		$\checkmark$	$\checkmark$
Neutrophil Count Change		$\checkmark$	$\checkmark$
Platelet Count Change		$\checkmark$	$\checkmark$

### Dataset description

In the proposed work, a dataset is used from a follow-up study conducted in the state of São Paulo in Brazil to identify the critical predictors of relapse in LLV in patients co-infected with Leishmania-HIV^10^. This is a binary classification dataset. The variable details and different distributions are reported in Supplementary Section 3 (Supplementary Table S3, Supplementary Table S4). The authors have made it available to the public with the research article. The original data set contains 627 patients, of which 56 died during follow-up and are not included in the analysis; a total of 571 patients’ follow-up data are used in this study. The data set consists of the patients’ sociodemographic, clinical, and laboratory findings. An initial analysis of the data set revealed that both male (63.40%) and female (36.60%) patients were included. The maximum number of patients is in the age group 0-4 years (30.42%), followed by the age groups 0-19 years and 5-9 years. The co-morbidities found in patients are pneumonia (0.87%), heart disease (2.1%), lung disease (2.62%), liver disease (1.74%), diabetes (1.22%), CNS disease (9.8%), solid malignancy (0.70%), urinary tract infection (0. 20%) and lymphoma/leukemia (1.22%). As part of the dataset, there were clinical signs such as jaundice (5.80%), severe paleness (53.10%), hepatomegaly (73.10%), splenomegaly (75.30%), lower extremity edema (0.90%), and hemorrhagic manifestation (1.6%). Therapy administered to patients included amphotericin B deoxycholate (3.90%), liposomal amphotericin (75.0%), and antimonites (20.30%). Laboratory data included information on blood cell counts, that is, white blood cells, neutrophils, platelets, and blood serum levels: creatinine, ALT, AST, alkaline phosphatase, albumin, gamma-GT, hematocrit, and hemoglobin. Of the 571 patients, 38 suffered from relapsed VL.

### ML methods for relapse prediction

The conventional CPH model, while widely used for relapse analysis, has certain limitations that can be effectively addressed by methods based on survival ML. One of the primary drawbacks of the CPH model is its reliance on the proportional hazards assumption, which posits that the hazard ratios between different groups are constant over time. This assumption can be unrealistic in complex medical scenarios where the risk of relapse may change dynamically over time, particularly in patients with co-infections such as HIV and visceral leishmaniasis. Additionally, the CPH model is typically linear in its predictors, which may not capture intricate, non-linear relationships in the data.

To address the above-mentioned problems, survival ML models have been utilized in relapse prediction in this work. Survival ML models such as RSF, SSVM, and XGBoost overcome these limitations by not requiring the proportional hazards assumption and by effectively modeling nonlinear interactions between predictors. RSF, for instance, can capture complex interactions and non-linear effects by building an ensemble of decision trees, each focused on different aspects of the data. SSVM can handle high-dimensional data and complex boundary definitions, making it more flexible in identifying patterns that the CPH model might miss. XGBoost further enhances the predictive power by iteratively redefining weak learners to improve the overall accuracy of the model. These methods provide more robust and flexible alternatives to the CPH model, improving predictive accuracy and providing deeper insights into the factors that influence relapse. The ML-based survival models are discussed hereafter.

### ML Model overview

#### Random Survival Forests (RSF)

RSF is an ensemble learning method specifically designed for survival and relapse analysis, extending the concept of random forests to handle right-censored survival data. RSF constructs multiple decision trees using bootstrap samples of the data, where each tree divides the data based on random subsets of predictors, optimizing survival differences between nodes. The aggregation of the results of all trees produces an overall survival function that provides robust predictions. Mathematically, the cumulative hazard function for a subject *i* the covariate $X_i$ is estimated by:

1 $$\begin{aligned} \overline{H}(t|X_i)= \frac{1}{B} \sum _{b=1}^B \overline{H}_b(t|X_i) \end{aligned}$$

where *B* is the number of trees in the forest and $\overline{H}_b(t|X_i)$ is the cumulative hazard function estimated by the *b*-th tree.

#### Support Vector Machine (SVM)

SVM is a powerful classification method that identifies the optimal hyperplane to separate different classes within the feature space. In survival analysis, the Survival Support Vector Machine (SSVM) is adapted to handle censored data. Key features of SSVM include the use of kernel functions to map data into higher-dimensional spaces, capturing complex relationships, and maximizing the margin between different classes, which leads to more robust and generalizable models. Additionally, SSVM incorporates techniques to manage censored survival data, ensuring accurate predictions. The decision function for SVM is mathematically expressed as:

2 $$\begin{aligned} f(x)=sign(w \cdot x+b) \end{aligned}$$

where *w* is the weight vector, *x* is the feature vector, and *b* is the bias term.

#### eXtreme Gradient Boosting (XGBoost)

XGBoost is a gradient boosting framework renowned for its efficiency and performance. It constructs an ensemble of weak learners, typically decision trees, by iteratively refining the model based on errors from previous iterations. The main components of XGBoost include combining the predictions of multiple weak models to form a robust predictive model, incorporating regularization techniques to prevent overfitting and enhance model generalizability, and its versatility in handling various data types and loss functions, making it suitable for different prediction tasks. The objective function for XGBoost is mathematically expressed as:

3 $$\begin{aligned} {\text{\L } }(\theta )= \sum _{i=1}^n l(y_i,\overline{y}_i)+ \sum _{k=1}^K \Omega (f_k) \end{aligned}$$

where *L* is the objective function, $\theta$ represents the set of all the model parameters that need to be optimized in XGBoost, *l* is a differentiable convex loss function that measures the difference between prediction $\overline{y}_i$ and the target $y_i$, *K* The number of trees (weak learners) in the ensemble $f_k$ is the *k*-th decision tree (weak learner) in the ensemble and $\Omega$ is a regularization term for the complexity of the model.

### Optimized CPH performance from the risk predictors

In this work, the performance of the conventional CPH model has also been optimized in the prediction of relapse of VL using the important risk factors identified by the explicable method, SHAP. The optimized CPH is used to derive the important statistics (HR and p-value), which are different compared to the previous results, and these statistics are closer to the actual scenario compared to the results of the conventional approach. In Algorithm 1, first, a univariate analysis is performed on the predictors. The predictors have been selected and categorized into three multivariate models depending on the analysis. In the next step, survival ML models were applied, and the evaluated model performance was based on the C index. To identify the risk predictors, the Shapley values are calculated using SHAP on the model prediction, and the top important predictors that influence the relapse predictions have been ranked. Thereafter, the predictors are sequentially added to the CPH model to get the optimal performance of the model. Table 2 contains information on the performance of the optimized CPH model used in this study for the relapse analysis.

**Algorithm 1:**
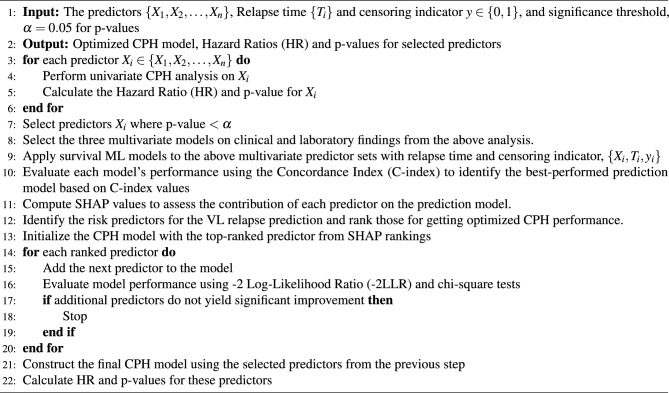
Relapse Prediction and Optimized CPH model from the Risk Predictors.

**Table 2:** CPH Model Performance Enhancement through Sequential Addition of Clinical Parameters Ranked by SHAP Values.

Clinical predictors	-2 Log Likelihood ratio	Degree of freedom	Significance of difference (p-value)
HIV	-41.08	1	
HIV+ Platelet count upon admission	-54.10	2	<0.001
HIV+ Platelet count upon admission + Change in Platelet count	-61.84	3	=0.005
HIV+ Platelet count upon admission + Change in Platelet count + Therapy with liposomal amphotericin	-74.22	4	<0.001
HIV+ Platelet count upon admission + Change in Platelet count + Therapy with liposomal amphotericin + Change in leucocyte count	-74.32	5	=0.75

The table presents the -2 log likelihood ratio (-2LLR) for each stepwise inclusion of predictors, starting from the topmost important feature identified by SHAP analysis in multivariate model 3, which is HIV, followed by subsequent additions of top-ranked features. The significance of the difference in -2LLR values was assessed using chi-squared tests, highlighting the contribution of each feature to the model’s performance. Results indicate that only the top four features significantly improve the conventional CPH model, emphasizing their importance as key clinical parameters.

## Results

### Experimental setup

Given the dataset’s significant class imbalance, with only 38 positive cases (less than 1%), the undersampling of the majority class was deemed unsuitable. To address this imbalance, the synthetic minority sample technique (SMOTE) has been used^52^, which synthetically oversamples the minority class, thus balancing the data set and improving the performance of the model. All experiments were implemented using Python 3.1 and the scikit-learn library, with hyperparameters detailed in Table 3. To ensure reliability and robustness, 10-fold cross-validation is employed in all experiments, and the average metrics from these iterations are reported to provide a comprehensive evaluation of the model’s performance. In designing the optimized CPH model, the key features identified by SHAP analysis have been incrementally added to achieve greater accuracy with minimal features, ensuring efficiency and interpretability. We also reported other results using 70:30, 80:20, and 90:10 dataset splits in Supplementary Section 4 (Supplementary Table S5). The dataset was split into training and validation sets. We have also used a stratified train-test split to ensure each class maintains the same distribution after splitting. Other observations, such as the loss curves, are reported in Supplementary Section 5 (Supplementary Figures S1, S2, S3, S4, S5, S6, S7, S8, and S9).

**Table 3:** Summary of predictive models for all the three multivariate clinical models in terms of their hyperparameters.

Statistical Model	Multivariate Model	Hyperparameters
RSF	Model 1	n_estimators=1000, min_samples_split=10, min_samples_leaf=15, n_jobs=-1
	Model 2	
	Model 3	
XGBoost	Model 1	eta=0.002, max_depth=3, objective=“survival:cox”, subsample=0.5, num_boost_round=1000, early_stopping_rounds=10, evals=[(dval, ‘val’)], verbose_eval=0
	Model 2	
	Model 3	
SSVM	Model 1	rank_ratio=0.1, max_iter=1000, tol=1e-6
	Model 2	rank_ratio=1, max_iter=1000, tol=1e-6
	Model 3	
CPH	Model 1	alpha=0.05
	Model 2	
	Model 3	

Whereas Model 1 contains all the clinical parameters, Model 2 includes all the laboratory parameters, and Model 3 includes both clinical and laboratory parameters.

### Relapse prediction analysis

**Fig. 2:**
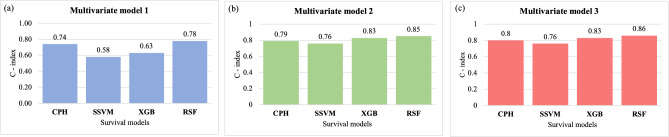
Comparison of the performance of different predictive models. RSF is found to be better performing than the conventional CPH model on the basis of c-index in all three multivariate models made with the factors related to VL relapse.

In this study, a comparative analysis of the proposed approach with the conventional CPH model of the existing article has been carried out, employing survival ML models in all multivariate models, based on the respective concordance indices (C-index). Upon analysis, it was observed that the RSF model consistently outperformed the CPH model in all three multivariate models (Fig. 2). Specifically, in Model 1, which included clinical parameters exclusively, the RSF model exhibited the highest performance with a C-index of 0.77, followed by the CPH model with a C-index of 0.74. Similarly, in Model 2, comprising solely laboratory parameters, the RSF model demonstrated superior performance with a C-index of 0.85, surpassing the CPH model’s C-index of 0.79.

### SHAP-based feature importance analysis

This study utilizes SHAP analysis to provide a comprehensive understanding of each feature’s role and how it contributes to the prediction outcomes of the survival ML model. SHAP, a commonly used method for understanding the importance of features in ML-based prediction, is based on the concepts of cooperative game theory. Using Shapley values ensures a fair distribution of importance. The SHAP analysis consists of the following steps: First, a background distribution is established as a reference for the model’s behavior. Then, multiple combinations of features are formed by examining all possible combinations. Finally, Shapley values are calculated to evaluate the average marginal contribution of each feature^53^. The values assist in determining the significance of each feature; high SHAP values imply a favorable contribution, while negative values suggest the opposite. Summary plots and dependency plots can be used to visualize SHAP values. Although SHAP analysis can not explain the underlying procedure of decision-making, this procedure strengthens the understanding of the impact of clinical features, enhancing the model’s interpretability and facilitating informed decision-making in clinical environments.

#### Interpretation of SHAP feature explanation

**Fig. 3:**
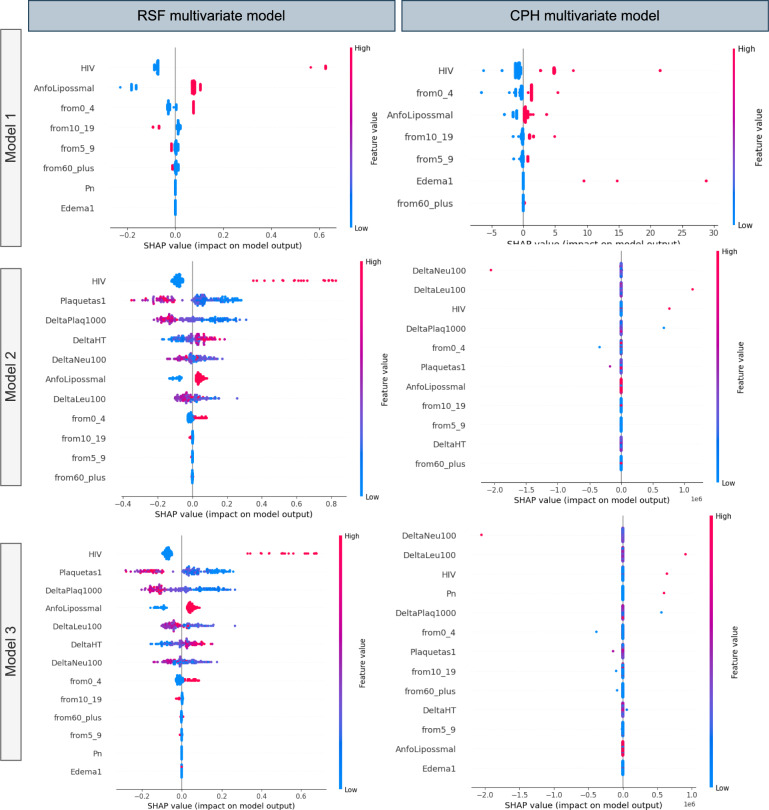
SHAP Summary plot illustrating the impact of different predictors of the multivariate models on the predicted survival models. The left column is for RSF, and the right column is for the CPH model of all three multivariate models. Each features or predictor are represented on the horizontal axis, where the color indicates the direction and magnitude of its effect on the predicted relapse probability. Features toward the top (in red) are associated with higher predicted relapse probabilities, while features toward the bottom (in blue) are associated with lower relapse probabilities.

Fig. 3 shows the SHAP summary plot that ranks different clinical characteristics based on their impact on output, which is VL relapse predicted by the respective survival models (RSF and CPH). The summary plot arranges the input variables along the y-axis based on their significance, while the SHAP value is shown along the x-axis. The strength of the dots is represented by their color, ranging from blue to red. The dots correspond to the cases in the dataset. The x-axis displays the range of predictions represented by the SHAP values for each variable, demonstrating the differences in the magnitudes of the input variables from blue to red. It can be used to gain a deeper understanding of how each unique attribute contributes to the prediction of the dependent variable. For example, when a feature has a high SHAP value and is positioned on the right side of the plot, it means that the feature has a substantial influence on the prediction of the dependent variable (VL relapse) when its magnitude is large. The summary plot combines feature importance with feature effects. Each point in the summary plot is a Shapley value for a feature and an instance. The bottom row of Fig. 3 shows the summary plot made from SHAP values calculated for RSF and CPH, respectively, trained on multivariate model 3. Multivariate model 3 consists of both laboratory and clinical findings, so it is better to compare the models trained on it. According to the best-performing prediction model, RSF (based on the c index), HIV co-infection has the highest impact on VL relapse, which was also reported in many previous studies^54^. In this figure, the red values on the left side and the blue values on the right side show that the presence of HIV co-infection leads to an increase in the risk of VL relapse, and absence leads to a decreased risk of VL relapse. After HIV, more impact of platelet count upon admission is found, and a change in platelet count is found on VL relapse. More blue color points in platelet count upon admission are on the right side of the lower platelet count, leading to an increase in the risk of VL relapse, and more red points of change in platelet count are on the left side reflecting an increase in the change in platelets, leading to an increased risk of VL relapse. Previous studies also found that lower platelet counts upon admission were associated with an increased risk of VL relapse in patients treated with antimony-based drugs^55^ and that platelet counts tended to decrease during active VL episodes and increase with successful treatment^56^.

Therapy with liposomal amphotericin is ranked after platelet count and platelet change, reflecting that it is the fourth feature of the dataset in order of its impact on the outcome. For liposomal amphotericin therapy, most of the red points on the right side show that most of the people who underwent liposomal amphotericin therapy experienced VL relapse. Liposomal amphotericin B is a commonly used treatment for VL due to its high efficacy and relatively low toxicity compared to other antileishmanial drugs. However, it is important to note that while liposomal amphotericin B is effective in treating VL, it may not eliminate the parasite from the body in all cases. The persistence of the parasite or the incomplete clearance could potentially lead to a relapse in VL symptoms. In addition, factors such as drug resistance, host immune status, and parasite strain variability may also influence treatment outcomes and the risk of relapse. The observed association between liposomal amphotericin therapy and relapsed LLV may reflect variations in treatment response, parasite clearance, or host immune factors between individuals receiving this therapy. However, despite its high efficacy, some studies have reported cases of relapse in VL after liposomal amphotericin B treatment^57^, highlighting the importance of continued follow-up and monitoring for patients treated with this drug.

RSF models excel in capturing nonlinear relationships and interactions, surpassing CPH models, which assume linearity between features and the log hazard. The ability of RSF to discern varied and pronounced effects on survival probabilities across different feature values results in a wider range of SHAP values. This contributes to the superior performance of RSF models, consequently leading to discrepancies in the generated summary plots between the two approaches. In Fig. 3 (multivariate model 3), which covers both clinical and laboratory parameters, changes in neutrophil and leukocyte counts are observed to exert a greater impact on VL relapse compared to HIV and pneumonia. In particular, platelet count changes and admission platelet count rank lower in the SHAP ranking produced by CPH. It is essential to emphasize that multivariate model 3 offers a comprehensive representation of an individual’s medical status, incorporating both clinical and laboratory parameters, whereas multivariate models 1 and 2 exclusively focus on clinical or laboratory findings, respectively. The first row of Fig. 3 depicts SHAP summary plots based on multivariate model 1 (clinical findings only) generated by RSF and CPH, respectively. Both plots underscore HIV as having the most significant impact on relapse of VL, consistent with previous studies^54^. Similarly, the second row ranks laboratory findings exclusively through SHAP summary plots. It reveals that among laboratory findings, HIV, changes in platelet count, and platelet count admission exert the most substantial influence on relapse of VL, as indicated by RSF and SHAP analysis. In contrast, according to CPH, changes in neutrophil count, changes in leukocyte count, and HIV exhibit more prominent effects on VL relapse.

#### Interpretation of SHAP dependence analysis

Dependence plots complement summary plots by providing a more detailed examination of the relationship between individual characteristics and model predictions. While summary plots offer an aggregate view of feature importance across the entire dataset, dependence plots allow for a granular analysis of how each feature influences predictions on a per-instance basis. This level of granularity is particularly valuable in understanding complex interactions between features and in identifying potential outliers or anomalies that may not be evident from summary statistics alone. Therefore, dependence plots serve as a crucial tool for gaining deeper insight into the workings of ML models and for validating model behavior against domain knowledge or expectations.

SHAP dependence plots serve as a useful tool for discerning the nuanced effects of individual features on an ML model’s predictions while simultaneously considering the influence of other features. These plots offer valuable insights into both the magnitude and directionality of a feature’s impact on the model’s output, thereby facilitating a deeper understanding and interpretation of complex ML models. The x-axis of the dependence plot represents the feature under investigation, while the y-axis depicts the associated SHAP values across different instances of that feature. Each data point on the plot corresponds to a specific instance, with its position indicating both the feature’s value and its corresponding SHAP value. Moreover, the color gradient strip adjacent to the plot provides visual cues regarding the interactions with another feature.

**Fig. 4:**
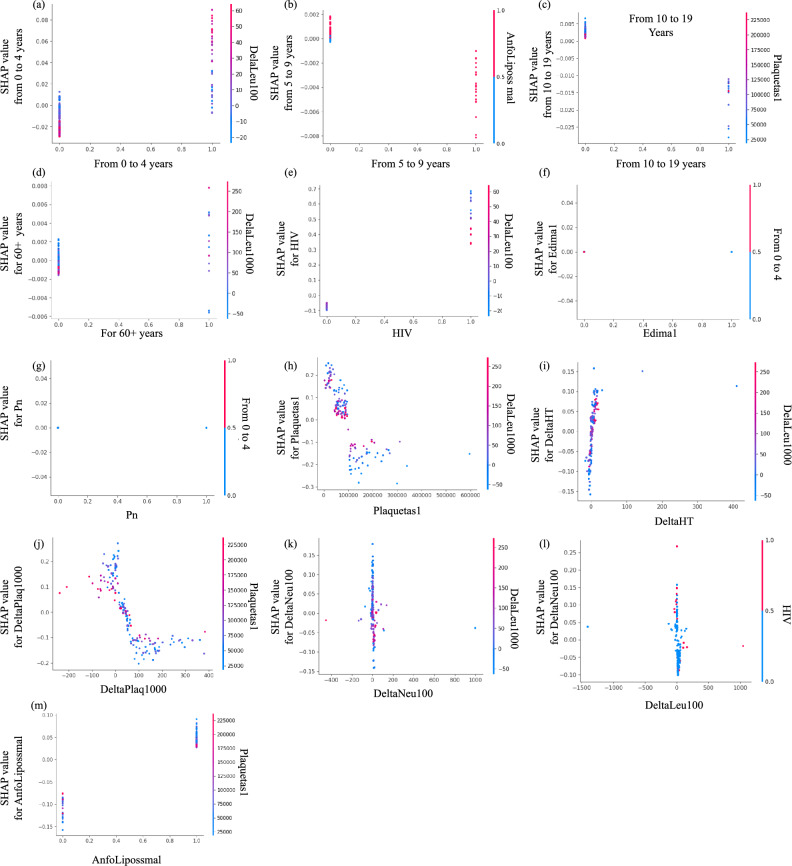
HAP dependence plot which explains the impact of single features on the model outcome for the predictors of multivariate model 3 that includes both the clinical and laboratory parameters, the SHAP values are calculated on the RSF model, which is the best-performing predictive model among all four models on the dataset.

Fig. 4 shows the dependence plot generated using the clinical parameters of multivariate model 3, incorporating clinical and laboratory findings. SHAP values were calculated based on the highest-performing survival model, namely RSF. In Figs. 4(a) to (d), the influence of various age groups on VL relapse is illustrated. In particular, Fig. 4(a) highlights that individuals aged 0-4 years have a greater impact on predicting the relapse of LLV compared to other age groups. Fig. 4(e) shows the interaction between HIV status and changes in platelet count during patient follow-up, revealing an increased impact of HIV positivity on VL relapse, along with notable fluctuations in platelet counts. The data set contains a limited number of cases concerning edema and pneumonia, as depicted in their respective dependence graphs (Figs. 4(f) and (g)), suggesting a minimal impact on predicting relapse of VL. Furthermore, Fig. 4(h) illustrates the interaction between platelet count and its variations, indicating that lower platelet counts exert a more pronounced effect on VL relapse, supported by positive SHAP values and greater platelet count variations between individuals with fewer platelets at admission; this was also shown in previous studies^10,55^ and the SHAP summary plot. Fig. 4(i) explores the interaction between changes in hematocrit and platelet count, emphasizing the increased impact of changes in hematocrit along with substantial variations in platelet count on relapse of LLV. Fig. 4(j) indicates that while an increase in platelet count variations can hinder the prediction of VL relapse, instances of significant changes in platelet count are infrequent in the dataset, with low platelet counts at admission correlated with an increased risk of VL relapse. Fig. 4(k) shows the interaction between changes in neutrophil count and platelet count, revealing that cases with low platelet counts often exhibit insignificant changes in neutrophil count. This plot further underscores the increased impact of low platelet counts on the prediction of VL relapse, contrasting with minimal effects on the prediction of VL relapse seen with changes in neutrophil count. Similarly, changes in leukocyte count (Fig. 4(l)) do not significantly impact the prediction of VL relapse. In particular, red data points indicating HIV presence contribute to positive SHAP values, signifying their impact on VL relapse, which is consistent with previous studies^10,54^. Finally, Fig. 4(m) illustrates the interaction between therapy involving liposomal amphotericin B and platelet count upon admission, suggesting a potential association between increased therapy use and VL relapse, consistent with trends observed in other dependence and summary graphs.

**Fig. 5:**
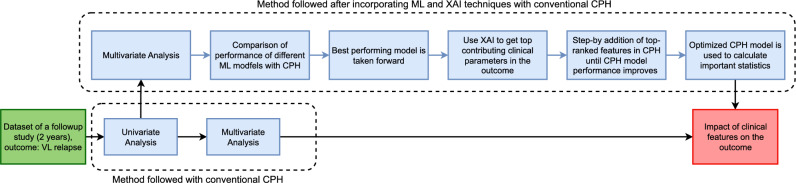
This figure highlighted the importance of incorporating ML techniques into the conventional approach of survival analysis using CPH model (approach 2). ML-based survival models and XAI techniques are utilized to get an optimized CPH, which is used to derive the important statistics (HR and p-value), which are different as compared to the previous results, and these statistics are closer to the actual scenario when compared to the results of the conventional approach.

### Optimized CPH performance

Furthermore, to validate the ML model and identify crucial clinical parameters, the characteristics have been sequentially integrated and ranked by the explainable ML model in the Cox regression model as depicted in Fig. 5. Beginning with the highest-ranking feature identified by SHAP analysis in multivariate model 3 (HIV), subsequent additions of top-ranked features were evaluated. The significance of the difference in the values of the logarithmic likelihood ratio -2 (-2LLR) was assessed using chi-square tests. The results indicated that only the top four characteristics (HIV, platelet count upon admission, change in platelet count, and liposomal amphotericin therapy) significantly improved the performance of CPH with a p-value less than 0.05 in the chi-square test, underscoring their importance as critical clinical parameters that XAI identifies. Table 4 contains the information on -2LLR and the chi-square test result when the CPH model was trained with the sequential addition of features in order of their ranking by SHAP in multivariate model 3. Table 4 shows the Hazard ratio (HR) and the associated p-value when the CPH model was used again with these four characteristics to make an optimum CPH model for such clinical settings. It suggests that the HR associated with HIV (HR=3.92, 95% CI=2.03-7.58) and platelet count upon admission (HR=1.00, 95% CI=1.00-1.00) are significant with a p-value less than 0.05. The change in platelet count (HR = 1.00, 95% CI = 0.99-1.00) and liposomal amphotericin B therapy (HR = 1.89, 95% CI = 0.99-1.00) do not have a statistically significant impact on VL relapse. This comparison shows that HIV has a significant relationship with VL relapse. People with HIV are more prone to VL relapse when adjusted for other clinical parameters chosen with the help of RSF and subsequently with the -2LLR test.

**Table 4:** The Cox regression results for predicting VL relapse are summarized.

Clinical predictors	Hazard Ratio (95% CI)	p-value
HIV	3.92 (2.03-7.58)	<0.05
Platelet count upon admission	1 (1.00-1.00)	0.02
Change in platelet count	1 (0.99-1.00)	0.13
Therapy with liposomal amphotericin	1.89 (0.89-49.15)	0.07

The table presents the hazard ratios and associated p-values for the top four features identified by the XAI tool, SHAP and significantly contributing to the model performance of CPH. HIV status emerges as a significant predictor with a hazard ratio of 3.92 (Previously, it was 7.47 (2.58–21.55) p-value <0.001) and a p-value of less than 0.005. Platelet count upon admission shows marginal statistical significance with a hazard ratio of 1 and a p-value of 0.02. Similarly, the change in platelet count does not have a statistically significant association with VL relapse, as indicated by its hazard ratio of 1 and a p-value of 0.13. Therapy with liposomal amphotericin exhibits a hazard ratio of 1.89, suggesting a nearly twofold increased risk of VL relapse, although the association is not statistically significant at the conventional threshold (p = 0.05), indicating a trend but not a definitive relationship with VL relapse.

## Discussion

### Domain interpretation and literature support

The SHAP summary plot results align well with established clinical knowledge regarding VL relapse in HIV co-infected individuals. HIV status, the most influential predictor in our model, has consistently been associated with increased relapse risk due to impaired immune response and reduced treatment efficacy^54,55^. Similarly, platelet count and its variations are well-known markers of hematologic dysfunction in VL, with low counts at admission and unstable trends during follow-up linked to poor prognosis^56^. The specific cases of relapses observed in the case of LLV treatment further strengthen the idea of variation in individual treatment response, resulting in incomplete parasite clearance owing to individual immune status, which might be suppressed as a result of HIV infection. This further highlights the importance of the model in predicting treatment outcome with liposomal formulation, particularly among high-risk groups suffering from VL-HIV co-infection^57^. These domain-aligned interpretations support the reliability of the model outputs and underscore the value of integrating machine learning with clinical insight.

### Time complexity

The main contributions to time complexity come from three main components of the method: CPH model fitting, multivalent ML models, and SHAP analysis. Fitting the CPH model involves maximizing the partial likelihood function, with a time complexity of $O(n \log n)$, where $n$ represents the number of observations. The calculation of HR and p-value has constant complexity. For multivariate ML models, the time complexity of SSVM is $O(n^3)$, where $n$ is the number of observations. The time complexity of XGBoost is $O(K \cdot n \cdot \log n)$, where $K$ is the number of boost rounds and $n$ is the number of observations, while RSF has a complexity of $O(T \cdot n \cdot \log n)$, where $T$ is the number of trees in the forest. Calculating exact SHAP values involves evaluating all possible combinations of features, with a time complexity of $O(2^m \cdot n \cdot T)$, where $m$ is the number of features, $n$ is the number of samples and $T$ is the time to evaluate the model. In addition, optimizing the CPH model is an iterative process that contributes to the overall complexity.

### Guideline to use the Pipeline

The pipeline consists of a conventional predictive model, namely CPH, and three ML models, along with a SHAP interpretation. It is noted that the interpretation is not automotive; it requires a human-in-loop concept, i.e., a healthcare professional is responsible for making the final decision. This is a tool that can demonstrate different interpretable statistics. A healthcare professional must understand ‘how to interpret different outputs’. Healthcare professionals are provided with different outcomes of different ML models and, hence, can understand the diversity of the outcomes. SHAP interpretation of different ML methods, such as feature (risk factor) importance and dependency analysis (co-relation), are provided. Healthcare professionals must verify the outcomes across different ML models to understand the risk and confidence. After identifying the important risk factors, healthcare professionals are free to use the specific risk factors to include in the final CPH predictive models. Although the proposed method suggests optimal CPH methods that include top-ranked risk factors, it is advised to use the interpretation of SHAP wisely. The TRIPOD+AI checklist related to the article is reported in Supplementary Table S1.

### Limitations

Despite the promising results, this study has several limitations that warrant consideration. To begin with, the hired dataset for model development and validation comprises follow-up data from only 571 patients, out of which no more than 38 VL relapse cases were cases. The low number of sample data, more specifically, the minimal number of VL relapse cases, could influence the statistical power of the models. Less power could neutralize their capability of capturing rare but important patterns in the data, so an event-to-predictor ratio of such a small value increases the risk of overfitting and thus, may decrease generalizability into broader patient populations. Secondly, the cohort is single-centered and located in São Paulo, Brazil, so it does not necessarily reflect the epidemiological diversity observed in other endemic VL locations. Even though survival machine learning methods such as RSF, SSVM, XGBoost, etc., enhance their prediction accuracy, their application of these models demands considerable computations, especially in the context of hyperparameter tuning and SHAP-based explainability analysis. The interpretability standpoint of SHAP values is valuable, but they can sometimes be misleading if taken out of context within clinical frameworks and result in overdependence on model outputs. Finally, their retrospective nature and limitation of following up to exclude any patients who died in critical situations may result in selection bias, probably underestimating the risk factors for severe outcomes with the disease.

### Concluding remarks

In conclusion, this study aimed to achieve three main objectives: first, to assess the performance of survival ML models against the conventional CPH model; second, to enhance the interpretability of these models (how the features i.e. risk factors are contributing and the co-relation) using an explainable method, SHAP and identify the crucial clinical parameters based on the ranking done by SHAP, in this case using SHAP summary plot; and third, to analyze the impact of top parameters identified by the best-performing ML model on the prediction, based on Shapley values and using those predictors make an optimum CPH which can provide us with important statistical measures which play an important role in relapse analysis. The findings underscored the superior performance of RSF over the CPH model in all multivariate settings. ML models exhibited a higher C-index than the traditional CPH model, indicating enhanced predictive capabilities. In particular, RSF consistently outperformed the CPH model, demonstrating its efficacy in capturing non-linear relationships and interaction effects between variables inherent in survival analysis. Furthermore, applying XAI techniques, particularly SHAP values, facilitated the interpretation of model predictions and identified useful characteristics that influence the outcome. The summary and dependence graphs generated using SHAP values provided valuable information on the relative importance of features in predicting the risk of relapse of VL. The clinical characteristics ranked by the summary plot were utilized to make an optimal CPH that can provide valuable information on the relationship of these characteristics with the outcome by computing statistical measures, i.e., HR and the corresponding p-value. Clinicians can use this information to prioritize important clinical and laboratory parameters, improving risk assessment and treatment decision-making. In the future, the focus will be on using similar techniques in datasets collected in India to conduct thorough analyses of infectious diseases of regional significance. As infectious diseases have regional significance, comprehensive studies are needed to understand the essential characteristics associated with the disease. By applying survival ML models and XAI techniques to India-specific datasets, insight can be gained into the factors influencing disease outcomes in this context. This could lead to the development of customized disease management and prevention approaches, ultimately improving public health outcomes in the region.

## Supplementary Information

Supplementary Information 1.

Supplementary Information 2.

## Data Availability

The dataset is available publicly in https://www.ijidonline.com/cms/10.1016/j.ijid.2020.02.028/attachment/65c465be-111a-40b5-8231-4392b6c06695/mmc1.xlsx. The pre-processed data is available in the supplementary.

## Code availability

The code can be obtained by requesting the corresponding author.
