# Supplementary material for: AI-driven analysis by identifying risk factors of VL relapse in HIV co-infected patients
Source: Sci Rep. 2025 Jul 1;15:21067. doi: 10.1038/s41598-025-07406-7 (PMC12215746; doi:10.1038/s41598-025-07406-7)
Supplement: Supplementary file 2 — Supplementary Information 2. [file 41598_2025_7406_MOESM2_ESM.pdf]

Supplementary: AI-driven Analysis by Identifying Risk Factors of VL Relapse in HIV Co-infected Patients

Abhishek Kumar[1], Sanchita Mondal[1], Debnarayan Khatua[2,+], Debashree Guha Adhya[1,+], Budhaditya Mukherjee[1,+], Arista Lahiri[3,+], Dilip K. Prasad[4], and Arif Ahmed Sekh[4,*]

[1]School of Medical Science and Technology, IIT Kharagpur, West bengal, 721302, India
[2]Department of Mathematics and Statistics, Vignan's Foundation for Science, Technology and Research, Andhra Pradesh, 522213, India
[3]Dr B C Roy Multi Speciality Medical Research Centre, IIT Kharagpur, West bengal, 721302, India
[4]Department of Computer Science, UiT The Arctic University of Norway, Tromsø, 9017, Norway
[*]arif.a.sekh@uit.no
[+]these authors contributed equally to this work

ABSTRACT

1 TRPOD+AI checklist

The TRIPOD+AI Checklist is a comprehensive guide for reporting clinical prediction models that use regression or machine learning methods. In our article, "AI-driven Analysis by Identifying Risk Factors of VL Relapse in HIV Coinfected Patients", we have incorporated the checklist to ensure that all critical aspects of model development and evaluation are transparently reported.

Table S1 is the TRPOD+AI[1] checklist of the article.

Table S1. TRIPOD+AI Checklist

Section/ topic	*Item*	*Development/ evaluation**	*Checklist item*	Our Work
**Title**				
**Title**	1	D;E	Identify the study as developing or evaluating the performance of a multivariable prediction model, the target population, and the outcome to be predicted	N/A
**Abstract**				
Abstract	2	D;E	See TRIPOD+AI for Abstracts checklist	N/A
**Introduction**				
Background	3a	D;E	Explain the healthcare context (including whether diagnostic or prognostic) and rationale for developing or evaluating the prediction model, including references to existing models	Reported (Page 1)
	3b	D;E	Describe the target population and the intended purpose of the prediction model in the context of the care pathway, including its intended users (eg, healthcare professionals, patients, public)	Reported (Page 2)
	3c	D;E	Describe any known health inequalities between sociodemographic groups	N/A
Objectives	4	D;E	Specify the study objectives, including whether the study describes the development or validation of a prediction model (or both)	Reported (Page 2,3)
**Methods**				
Data	5a	D;E	Describe the sources of data separately for the development and evaluation datasets (eg, randomised trial, cohort, routine care or registry data), the rationale for using these data, and representativeness of the data	Reported (Page 4,5)
	5b	D;E	Specify the dates of the collected participant data, including start and end of participant accrual; and, if applicable, end of follow-up	Public Dataset (Page 15)
Participants	6a	D;E	Specify key elements of the study setting (eg, primary care, secondary care, general population) including the number and location of centres	Public Dataset (Page 15)
	6b	D;E	Describe the eligibility criteria for study participants	N/A
	6c	D;E	Give details of any treatments received, and how they were handled during model development or evaluation, if relevant	N/A
Data preparation	7	D;E	Describe any data pre-processing and quality checking, including whether this was similar across relevant sociodemographic groups	Reported (Page 4,5)
Outcome	8a	D;E	Clearly define the outcome that is being predicted and the time horizon, including how and when assessed, the rationale for choosing this outcome, and whether the method of outcome assessment is consistent across sociodemographic groups	Partially reported (Page 5,8,12,13)
	8b	D;E	If outcome assessment requires subjective interpretation, describe the qualifications and demographic characteristics of the outcome assessors	N/A
	8c	D;E	Report any actions to blind assessment of the outcome to be predicted	N/A
Predictors	9a	D	Describe the choice of initial predictors (eg, literature, previous models, all available predictors) and any pre-selection of predictors before model building	Reported (Page 4,5)
	9b	D;E	Clearly define all predictors, including how and when they were measured (and any actions to blind assessment of predictors for the outcome and other predictors)	Reported (Page 5,6, Sup-5,6)
	9c	D;E	If predictor measurement requires subjective interpretation, describe the qualifications and demographic characteristics of the predictor assessors	N/A

Section/ topic	***Item***	***Development/ evaluation****	***Checklist item***	Our Work
Sample size	10	D;E	Explain how the study size was arrived at (separately for development and evaluation), and justify that the study size was sufficient to answer the research question. Include details of any sample size calculation	Reported
Missing data	11	D;E	Describe how missing data were handled. Provide reasons for omitting any data	Rported (Page 4,5)
Analytical methods	12a	D	Describe how the data were used (eg, for development and evaluation of model performance) in the analysis, including whether the data were partitioned, considering any sample size requirements	Reported (Page 4,5,8)
	12b	D	Depending on the type of model, describe how predictors were handled in the analyses (functional form, rescaling, transformation, or any standardisation)	Reported (Page 7,8)
	12c	D	Specify the type of model, rationale†, all model building steps, including any hyperparameter tuning, and method for internal validation	Reported (Page 7,8)
	12d	D;E	Describe if and how any heterogeneity in estimates of model parameter values and model performance was handled and quantified across clusters (eg, hospitals, countries). See TRIPOD-Cluster for additional considerations‡	N/A
	12e	D;E	Specify all measures and plots used (and their rationale) to evaluate model performance (eg, discrimination, calibration, clinical utility) and, if relevant, to compare multiple models	N/A
	12f	E	Describe any model updating (eg, recalibration) arising from the model evaluation, either overall or for particular sociodemographic groups or settings	N/A
	12g	E	For model evaluation, describe how the model predictions were calculated (eg, formula, code, object, application programming interface)	Reported (Page 7,8)
Class imbalance	13	D;E	If class imbalance methods were used, state why and how this was done, and any subsequent methods to recalibrate the model or the model predictions	Reported (Page 7,8)
Fairness	14	D;E	Describe any approaches that were used to address model fairness and their rationale	N/A
Model output	15	D	Specify the output of the prediction model (eg, probabilities, classification). Provide details and rationale for any classification and how the thresholds were identified	Reported (Page 7,8)
Training versus evaluation	16	D;E	Identify any differences between the development and evaluation data in healthcare setting, eligibility criteria, outcome, and predictors	Reported (Page 5-8)
Ethical approval	17	D;E	Name the institutional research board or ethics committee that approved the study and describe the participant informed consent or the ethics committee waiver of informed consent	NA
**Open science**				
Funding	18a	D;E	Give the source of funding and the role of the funders for the present study	Reported (Page 14)
Conflicts of interest	18b	D;E	Declare any conflicts of interest and financial disclosures for all authors	Reported (Page 14)
Protocol	18c	D;E	Indicate where the study protocol can be accessed or state that a protocol was not prepared	N/A
Registration	18d	D;E	Provide registration information for the study, including register name and registration number, or state that the study was not registered	N/A
Data sharing	18e	D;E	Provide details of the availability of the study data	Reported (Page 15)
Code sharing	18f	D;E	Provide details of the availability of the analytical code§	Reported (Page 15)
**Patient and public involvement**				
Patient and public involvement	19	D;E	Provide details of any patient and public involvement during the design, conduct, reporting, interpretation, or dissemination of the study or state no involvement	N/A

Section/ topic	*Item*	*Development/ evaluation**	*Checklist item*	Our Work
**Result**				
Participants	20a	D;E	Describe the flow of participants through the study, including the number of participants with and without the outcome and, if applicable, a summary of the follow-up time. A diagram may be helpful	N/A
	20b	D;E	Report the characteristics overall and, where applicable, for each data source or setting, including the key dates, key predictors (including demographics), treatments received, sample size, number of outcome events, follow-up time, and amount of missing data. A table may be helpful. Report any differences across key demographic groups	N/A
	20c	E	For model evaluation, show a comparison with the development data of the distribution of important predictors (demographics, predictors, and outcome)	Reported (Page 8, Sup-5,6)
Model development	21	D;E	Specify the number of participants and outcome events in each analysis (eg, for model development, hyperparameter tuning, model evaluation)	Reported (Page 4,5, 8, Sup-5,6)
Model specification	22	D	Provide details of the full prediction model (eg, formula, code, object, application programming interface) to allow predictions in new individuals and to enable third party evaluation and implementation, including any restrictions to access or reuse (eg, freely available, proprietary)¶	Reported (Page 5-8, Page 14-15)
Model performance	23a	D;E	Report model performance estimates with confidence intervals, including for any key subgroups (eg, sociodemographic). Consider plots to aid presentation	N/A
	23b	D;E	If examined, report results of any heterogeneity in model performance across clusters. See TRIPOD-Cluster for additional details‡	N/A
Model updating	24	E	Report the results from any model updating, including the updated model and subsequent performance	N/A
**Discussion**				
Interpretation	25	D;E	Give an overall interpretation of the main results, including issues of fairness in the context of the objectives and previous studies	Reported (Page 8-13)
Limitations	26	D;E	Discuss any limitations of the study (such as a non-representative sample, sample size, overfitting, missing data) and their effects on any biases, statistical uncertainty, and generalisability	Reported (Page 14)
Usability of the model in the context of current care	27a	D	Describe how poor quality or unavailable input data (eg, predictor values) should be assessed and handled when implementing the prediction model	N/A
	27b	D	Specify whether users will be required to interact in the handling of the input data or use of the model, and what level of expertise is required of users	N/A
	27c	D;E	Discuss any next steps for future research, with a specific view to applicability and generalisability of the model	Reported (Page 14)

2 Abbreviations

Table S2 is the abbreviation list used in the article.

Table S2. Abbreviations

Sl. No.	Abbreviation	Full Form
1	VL	Visceral Leishmaniasis
2	RSF	Random Survival Forest
3	SSVM	Survival Support Vector Machine
4	XGBoost	eXtreme Gradient Boosting
5	CPH	Cox Proportional Hazard
6	ML	Machine Learning
7	SHAP	SHapley Additive exPlanation
8	HIV	Human immunodeficiency viruses
9	XAI	eXplainable Artificial Intelligence
10	ISC	Indian subcontinent
11	PKDL	Post-Kala-Azar-Dermal-Leishmaniasis
12	ART	Antiretroviral Therapy
13	cART	Combined Antiretroviral Treatment
14	GradCAM	Gradient-weighted Class Activation Mapping
15	CNN	convolutional neural network
16	LIME	Local Interpretable Model-agnostic
17	EBM	Explainable Boost Machin
18	CBR	Case-Based Reasonin
19	SMOTE	Synthetic Minority Sample Technique

3 Demographic Information and Variable Details of the Dataset

The dataset in our study includes several categorical variables that are crucial to analyzing the risk factors of relapse of VL in HIV-coinfected patients. These variables are pneumonia, which indicates the presence or absence of pneumonia; liposomal amphotericin, which indicates whether the patient received liposomal amphotericin treatment; HIV status, which indicates whether the patient is HIV positive or negative; edema, which indicates the presence or absence of edema; and relapse event, which indicates whether the patient experienced a relapse event. These variables provide essential information for building and evaluating our prediction models, helping us to understand the factors that can influence the risk of relapse of VL. Table S3 reports the demographic information and other details of the dataset. Table S4 reports the different variables and types.

Table S3. Demographic and other distribution of the dataset

Categorical Variable	Negative Count	Positive Count
Pneumonia	618	9
liposomalamphotericin	143	484
HIV	544	83
Edema1	622	5
relapseevent	588	39

Table S4. Variable name and type

Variable	Type
Pneumonia	Categorical Variables
liposomalamphotericin	Categorical Variables
HIV	Categorical Variables
Edema1	Categorical Variables
relapse event	Categorical Variables
Hb1	Continuous Variables
Ht1	Continuous Variables
plateletsonadmin	Continuous Variables
Plateletchange	Continuous Variables
relapsetime	Continuous Variables

4 Results of Different Data Split

Table S5 demonstrates the c-index of different methods varying different split criteria.

Table S5. Results (c-index) of different methods that vary the split options.

**Method**	**CPH**			**SSVM**			**XGB**			**RSF**		
**Split**	70|30	80|20	90|10	70|30	80|20	90|10	70|30	80|20	90|10	70|30	80|20	90|10
Model 1	0.71	0.73	0.81	0.51	0.58	0.72	0.65	0.68	0.57	0.81	0.85	0.80
Model 2	0.69	0.75	0.82	0.67	0.76	0.73	0.73	0.85	0.84	0.79	0.87	0.83
Model 3	0.69	0.76	0.83	0.68	0.77	0.74	0.72	0.85	0.84	0.79	0.87	0.82

5 Error/Loss Curve

Here we present different losses and other results observed during the training process.

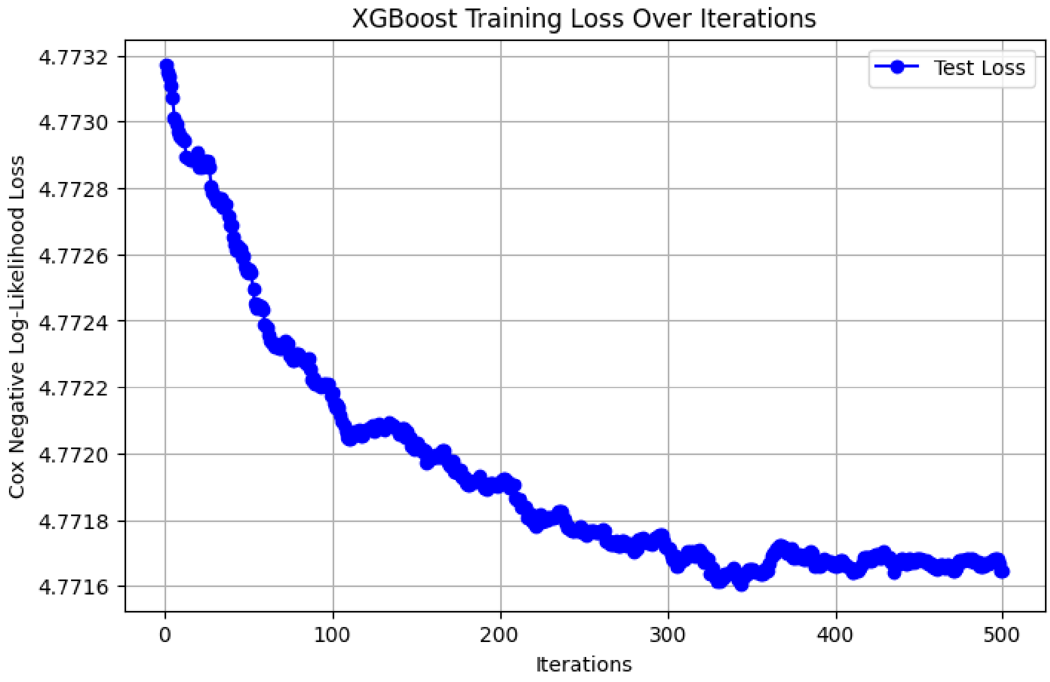

Figure S1. Model 1 XGB

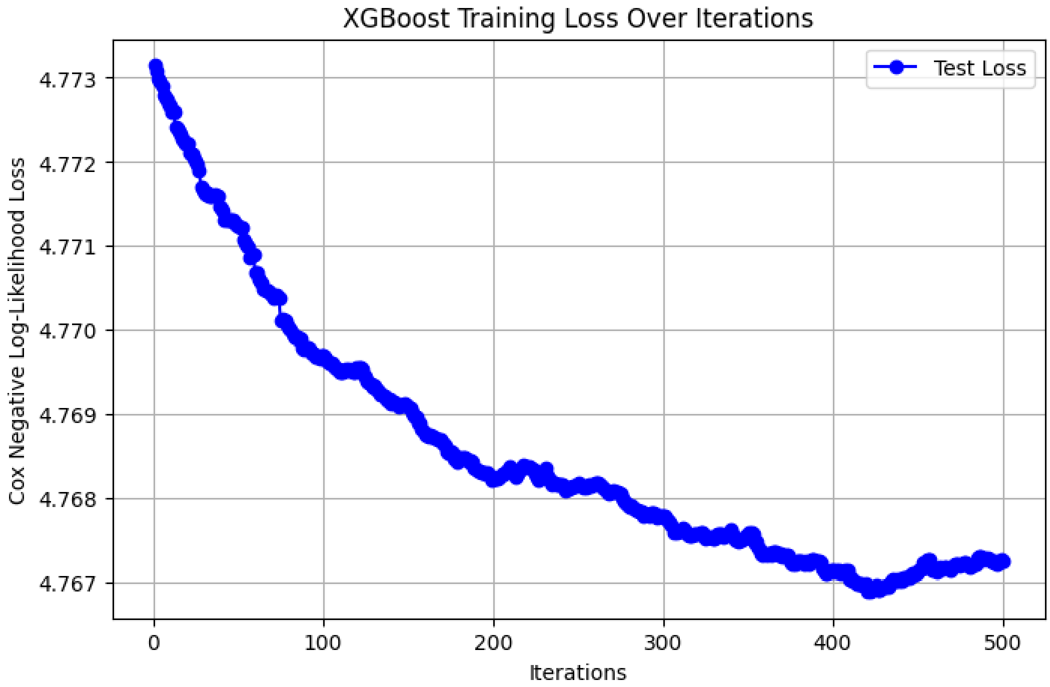

Figure S2. Model 2 XGB

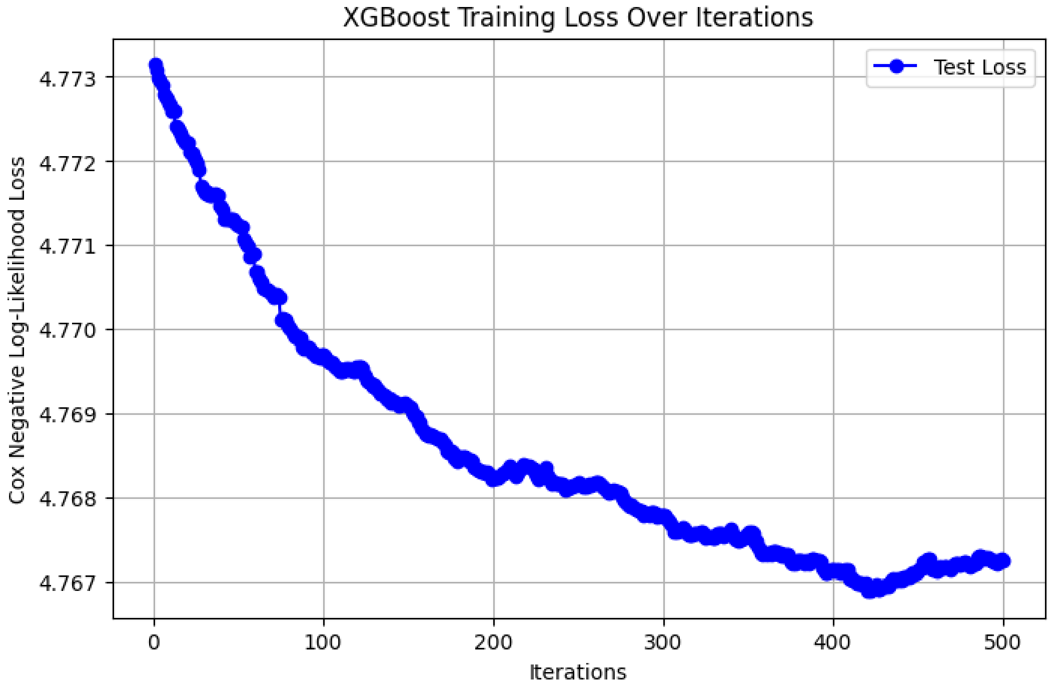

Figure S3. Model 3 XGB

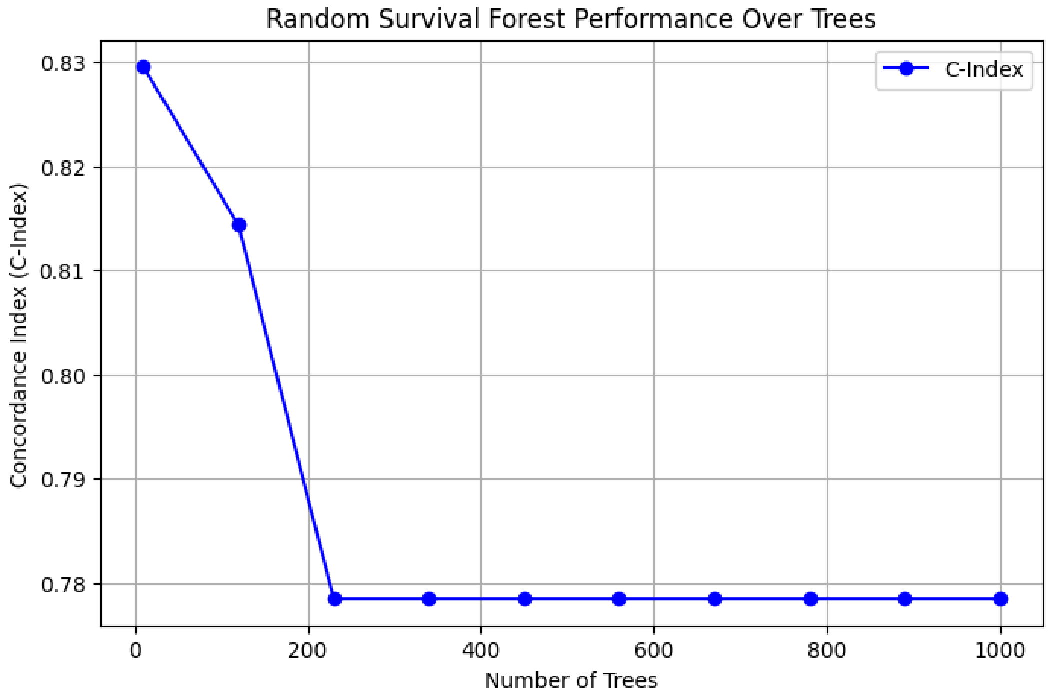

Figure S4. Model 1 RSF

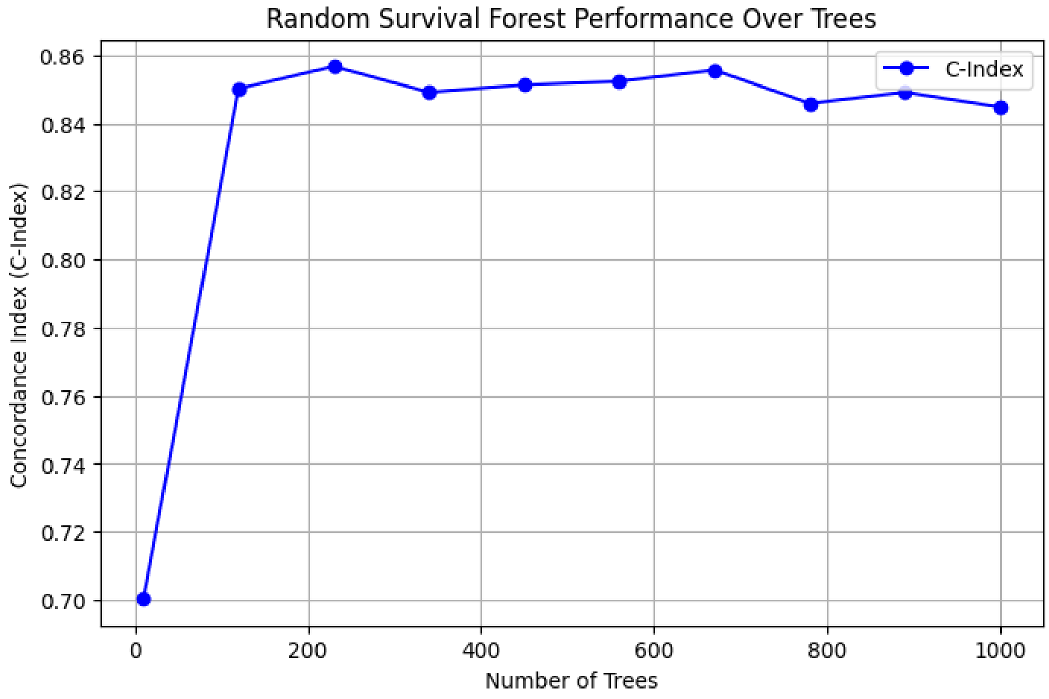

Figure S5. Model 2 RSF

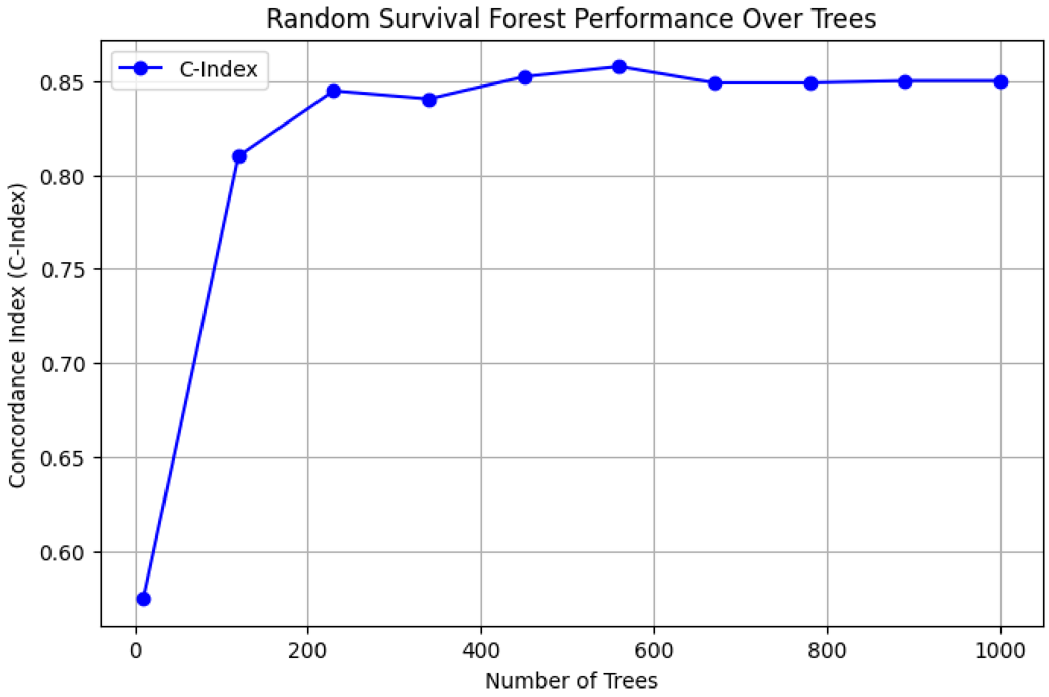

Figure S6. Model 3 RSF

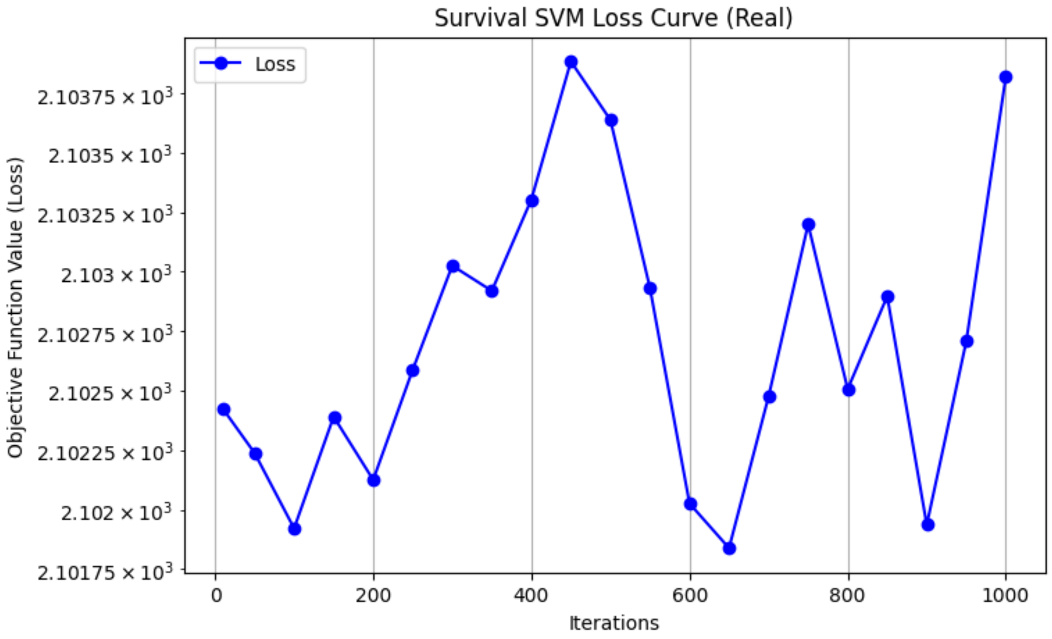

Figure S7. Model 1 SSVM

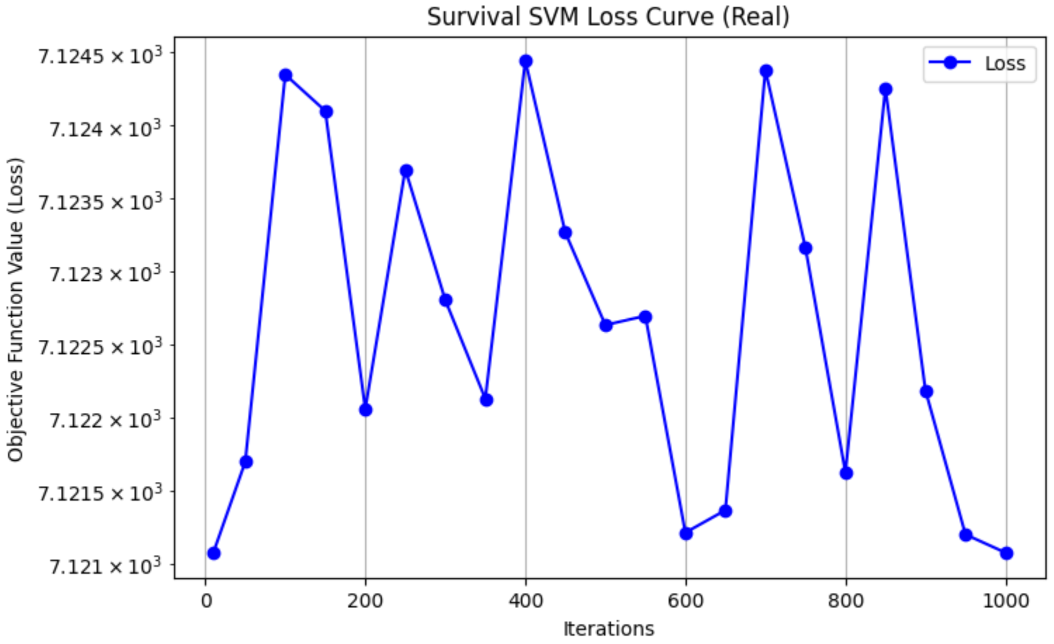

Figure S8. Model 2 SSVM

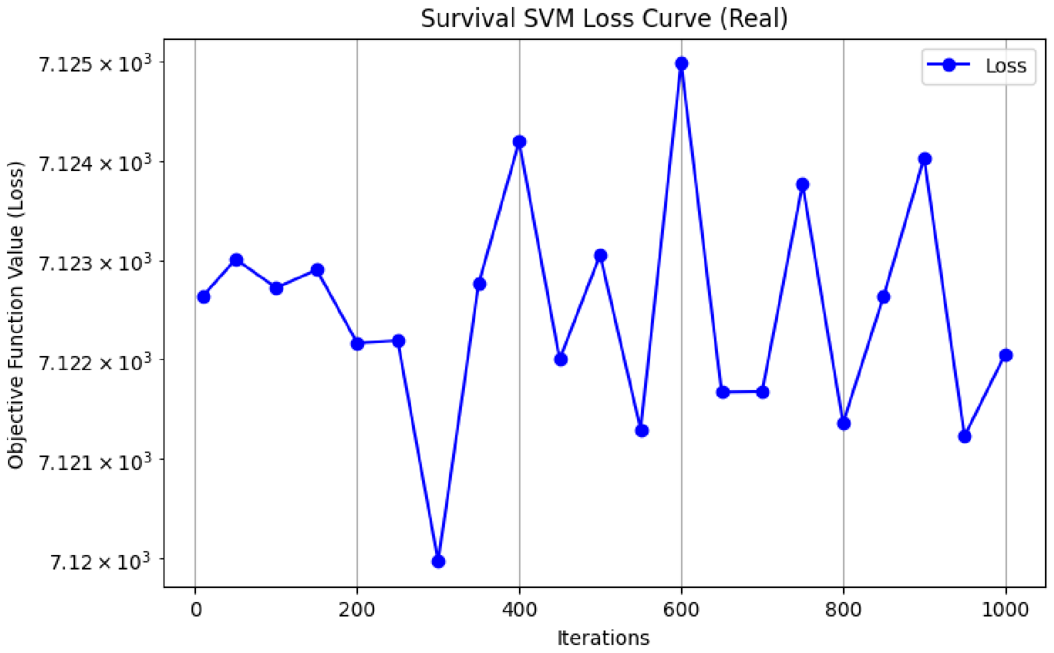

Figure S9. Model 3 SSVM

References

1. Collins, G. S. *et al.* Tripod+ ai statement: updated guidance for reporting clinical prediction models that use regression or machine learning methods. *bmj* **385** (2024).
